# Trends in Child Immunization across Geographical Regions in India: Focus on Urban-Rural and Gender Differentials

**DOI:** 10.1371/journal.pone.0073102

**Published:** 2013-09-04

**Authors:** Prashant Kumar Singh

**Affiliations:** International Institute for Population Sciences, Mumbai, India; Aga Khan University, Pakistan

## Abstract

**Background:**

Although child immunization is regarded as a highly cost-effective lifesaver, about fifty percent of the eligible children aged 12–23 months in India are without essential immunization coverage. Despite several programmatic initiatives, urban-rural and gender difference in child immunization pose an intimidating challenge to India’s public health agenda. This study assesses the urban-rural and gender difference in child immunization coverage during 1992–2006 across six major geographical regions in India.

**Data and Methods:**

Three rounds of the National Family Health Survey (NFHS) conducted during 1992–93, 1998–99 and 2005–06 were analyzed. Bivariate analyses, urban-rural and gender inequality ratios, and the multivariate-pooled logistic regression model were applied to examine the trends and patterns of inequalities over time.

**Key Findings:**

The analysis of change over one and half decades (1992–2006) shows considerable variations in child immunization coverage across six geographical regions in India. Despite a decline in urban-rural and gender differences over time, children residing in rural areas and girls remained disadvantaged. Moreover, northeast, west and south regions, which had the lowest gender inequality in 1992 observed an increase in gender difference over time. Similarly, urban-rural inequality increased in the west region during 1992–2006.

**Conclusion:**

This study suggests periodic evaluation of the health care system is vital to assess the between and within group difference beyond average improvement. It is essential to integrate strong immunization systems with broad health systems and coordinate with other primary health care delivery programs to augment immunization coverage.

## Introduction

India accounts for the highest number of under-five deaths in the world [1]. The average annual rate of decline in under-five mortality at 3.1% during 1990–2009 was considered insufficient to achieve the fourth Millennium Development Goal (MDG) of minimizing under-five mortality to 39 per 1000 live births by 2015 [2]. Globally, vaccine preventable diseases account for nearly 20% of all deaths occurring annually among children under five years of age, and immunization has a vital role to play in achieving the goals specified in the Millennium Declaration [3]. An assessment of the global measles mortality shows that India accounted for nearly 47% of the estimated measles mortality in 2010, followed by the World Health Organisation (WHO) Africa region at 36% [4]. Further, it has been estimated that more than half of all child deaths in India can be averted with interventions that are effective and widely practicable [5].

Since the launch of the Expanded Program on Immunization (EPI) by the WHO in 1974, the effectiveness of immunization has been thoroughly proven. Unlike most other health and development interventions, immunization does not just raise the chances that children will resist a disease, it virtually guarantees they will [6], [7]. Moreover, child immunization has been established worldwide as a highly cost-effective lifesaver [8], [9]. In 2005, WHO and United Nations Children’s Fund (UNICEF) developed the Global Immunization Vision and Strategy (GIVS), with the aim of reducing vaccine-preventable disease related morbidity and mortality by improving national immunization programs [10]. However, according to the Global Routine Vaccination Coverage (GAVI)–2010, about 19.3 million children were not fully vaccinated and remained at risk for diphtheria, tetanus, and pertussis and other vaccine-preventable causes of morbidity and mortality, and about 50% of these children are from India, Nigeria, and Congo [11]. Even though the immunization services in India are being offered free of cost in public health facilities, about 45% of Indian children are deprived of the recommended vaccinations [12].

After independence in 1947, it took three decades for India to articulate its first official policy for childhood vaccination; nevertheless, childhood immunization has been an important part of the Reproductive and Child Health (RCH) services [9], [13]. In line with the WHO goal, ‘Health for All by 2000’, the Indian government launched the Universal Immunization Program (UIP) in 1985 and embarked on a mission to achieve immunization coverage of all children [14], [15]. Since the inception of the immunization program, the overall policy and financial decisions have been taken at the national level, but the implementation is decided at the state level [13].

India, with a population of over 1.21 billion, exhibits one of the highest socioeconomic and demographic heterogeneities ever experienced anywhere in the world at the regional level [16]. There is a considerable evidence of marked regional and socioeconomic inequities in child health and mortality [5], [17]–[20]. A clear regional gap in key socioeconomic and development indicators across six major geographical regions is highlighted in **Table 1**. These differences in indicators are primarily the outcome of differences in community-level development, population composition, state health expenditure, poverty levels, status of women, and availability, accessibility and affordability of maternal and child health care services and their utilization [19], [21]–[25].

**Table 1:** Socioeconomic and demographic profile of population across six major geographical regions# in India.

Indicators	North	Central	East	Northeast	West	South	India
Population in million^a^	166	292	267	46	175	266	1210
Population share^a^ (in %)	13.7	24.1	22.1	3.8	14.5	22.0	−
Urbanization^a^	28.6	22.1	18.8	15.7	40.8	34.4	27.8
Female illiteracy^a^	53.0	63.1	60.3	36.0	30.2	28.7	50.1
Percentage of Muslim population^a^	11.3	14.1	16.9	22.8	10.0	11.1	13.4
Percentage of Scheduled Castes/Scheduled Tribes population^a^	11.3	26.6	27.0	33.3	19.9	20.3	24.4
Percentage of population in the poorest wealth quintile^b^	15.6	33.0	37.0	22.1	11.8	10.8	24.8
Mean age at first marriage^b^	17.5	16.6	16.2	18.1	17.6	18.0	17.1
Mean children ever born^b^	3.1	3.7	3.3	3.0	2.6	2.3	3.1
Percentage of currently married women using modern contraceptivemethods^b^	38.8	26.8	26.6	22.9	46.5	55.0	35.4
Percentage of women do not have any exposure of mass media^b^	36.4	37.8	46.0	28.8	26.6	15.1	33.9
Percentage of women utilized full antenatal care^b^	55.1	32.1	36.5	44.8	73.7	88.3	52.4
Percentage of women utilized skilled birth attendance^b^	37.9	21.5	27.2	27.0	60.6	74.1	38.8

Note:

# Excluded Union Territories and Islands.

a Provisional population totals 2011.

b National Family Health Survey, 2005–06.

Modern contraceptive method includes female sterilization, male sterilization, pill, IUD, injectable, condom.

Full ANC includes those women who had had a minimum of three antenatal visits, at least two tetanus toxoid injections during pregnancy or received one tetanus toxoid injection during pregnancy and at least one in the three years prior to the pregnancy, and received iron and folic acid tablets for 90 days or more.

Skilled birth attendance includes delivery conducted either in a medical institution, or home delivery assisted by a doctor/nurse/lady health visitor (LHV)/auxiliary nurse midwife (ANM)/other health professional.

One of the most consistent and significant findings in public heath literature is the strong association between place of residence [21], [26], and gender [27]–[30] on child health, net of other factors. The urban health advantage has often been attributed to the improved modern health care system that facilitates public health interventions [31]. Additionally, urban areas offer more choices, including greater availability of food, housing and health services [32]. Improved electricity, transportation, water and sanitation services are also, on average, more widely available in urban areas than in rural [31]. Besides, factors that determine health status differ between urban and rural areas significantly [33].

While, in general, the urban-rural difference in health is a phenomenon of developing countries and countries in transition, the gender gap in health and mortality is typically endured in South Asian countries. According to the recent Global Gender Gap Report (2011), India ranked 113 out of 135 countries in the overall Global Gender Gap Index, but ranked even lower (134) in the health and survival category [34]. Evidences of gender bias in health, survival, education and work participation in South Asian countries in general, and India in particular, are well documented in literatures [28], [35]–[38]. Scholars argue that the persisting gender inequality in health and other indicators in entire South Asian countries is the result of historic negligence – women are less likely to be literate, continue in their education, participate in the labour force, receive equal pay for similar work, and hold a political position than men [39].

Globally, monitoring of inequalities in health is foremost in the agenda of public health surveillance [40]–[42]. Recently, Ministers of Health from 194 countries at the 65th World Health Assembly held in Geneva endorsed the Global Vaccine Action Plan (GVAP), a roadmap to prevent millions of deaths by 2020 through equitable access to vaccines for people in all communities [43]. Addressing regional and socioeconomic differences in health and encouraging equitable distribution of health services has been one of the prime goals of India’s contemporary health policy [44]. In order to achieve this goal, an assessment of health outcomes across space, time and group is desirable. There is little evidence that highlights the regional progression in child immunization coverage by place of residence (urban-rural) and gender (male-female) in India. Thus, the present study is a modest approach in this direction.

## Data and Methods

### Ethics Statement

This study is based on the data available in the public domain; therefore, no ethics statement is required for this work.

### Data

The present study used data from three rounds of the National Family Health Survey (NFHS), the Indian version of the Demographic and Health Survey (DHS) conducted during 1992–93, 1998–99 and 2005–06 [45]–[47]. All the three rounds of the survey are nationally representative and have covered more than 99% of India’s population. The NFHS-1 covered a sample of 89,777 ever-married women aged 13–49, NFHS-2 covered 90,303 ever-married women aged 15–49 and NFHS-3 covered 124,385 women aged 15–49. The principal objective of NFHS is to provide state and national level estimates of fertility, mortality, family planning, HIV-related knowledge, and important aspects of nutrition, health and health care. The survey provides state and national level estimates of demographic and health parameters as well as data on various socioeconomic and program dimensions. All three rounds of NFHS have adopted similar sampling schemes and used similar interview schedules. The household and eligible female informant response rates were consistently above 90% in all three NFHS rounds. The details of the sampling weights are given in NFHS reports of the various rounds [45]–[47].

### Defining Outcome Variable and Sample Size

The outcome variable of the study is the full immunization among children aged 12–23 months. According to the guidelines developed by the WHO, children are considered fully immunized when they receive vaccination against tuberculosis (BCG), three doses of diphtheria, whooping cough (pertussis), and tetanus (DPT) vaccine; three doses of poliomyelitis (polio) vaccine and one dose of the measles vaccine by the age of 12 months. BCG should be given at birth or at first clinical contact, DPT and polio require three vaccinations at approximately 4, 8, and 12 weeks of age, and the measles vaccine should be given at age 12 months or soon after reaching 9 months of age [47].

Information on child immunization was collected from the immunization cards shown by household members during the survey or in the absence of the immunization card, the information gathered from the mother of the respective child was recorded. This is the standard procedure that the NFHS (or DHS) adopts to collect information on immunization coverage in other developing or underdeveloped countries. As the information on child immunization in NFHS-1 (3 live births in last four years), NFHS-2 (2 live births in last three years), and NFHS-3 (5 live births in last five years) was not along similar lines, thus the present analysis is restricted to the last two live births in three years preceding the survey to make the estimates comparable. The analytical sample size of the present study is restricted to 11596, 9484 and 9521 children aged 12–23 months in NFHS-1, NFHS-2, and NFHS-3 respectively.

### Defining Predictor Variables

To examine the net effect of urban-rural and gender difference in child immunization during 1992–2006, socioeconomic and demographic predictors were included in the analysis. These indicators are based on their theoretical and observed importance applied in literature and availability of information in all three rounds of survey for better comparability. Socioeconomic and demographic predictors such as birth order & interval, status of the child, age of the woman at childbirth, mother’s education, mother’s working status, father’s education, religion, social group, mass media exposure, and household wealth were included as predictor variables in the study. The birth order of children and the interval between the childbirths were grouped as first birth order, birth order 2/3 & interval≤24 months and, birth order 2/3 & interval>24 months. Mother’s age at childbirth was categorized into 15–24, 25–34 and 35–49 years. The educational levels of the mother and father were defined using years of schooling and they were grouped into illiterate, literate but below primary, primary but below middle school, middle but below high school, and high school and above. The religion of the mother was categorized as ‘Hindu’, ‘Muslim’, and ‘Others (Sikh, Christians, Buddhist and others)’. Identification of the social group was based on the women’s self-reporting as ‘Others’, ‘Scheduled Castes’ and ‘Scheduled Tribes’. Mass media exposure has been assessed by considering how often the respondents read the newspaper, listen to the radio and watch television or cinema. Similarly, a relative index of household wealth was also calculated from the standard set of assets owned by the household, which included ownership of consumer items and dwelling characteristics. Individuals were ranked on the basis of their household scores and divided into different quintiles, each representing 20 percent of the score, between 1 (poorest) and 5 (wealthiest).

### Analytical Approach

**Figure 1:**
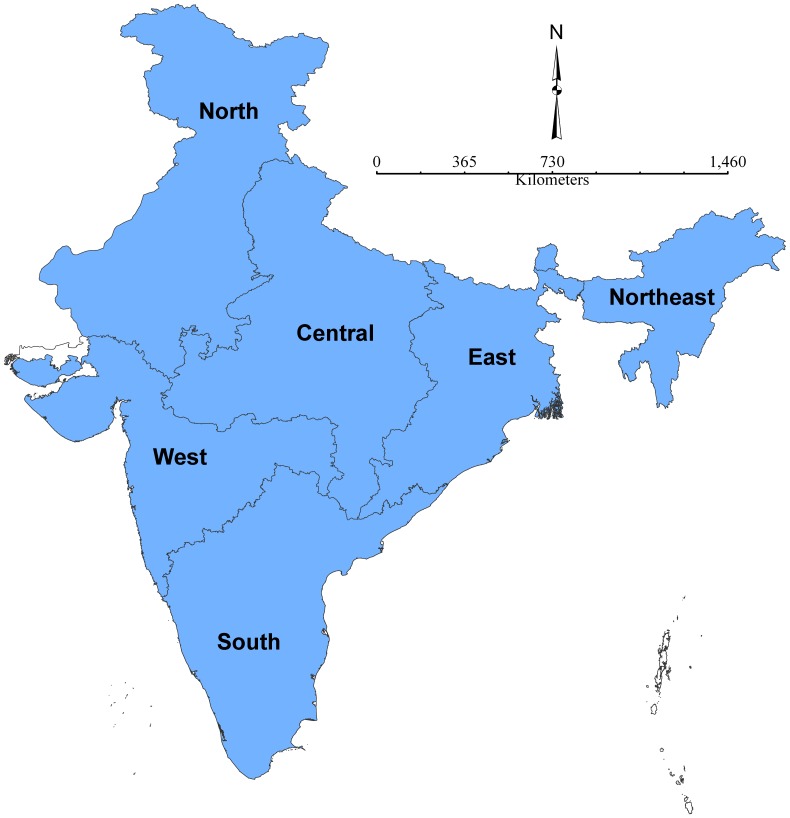
Location of Geographical Regions of India.

This study focuses on the urban-rural and gender differentials in full immunization coverage among children with respect to their region of residence. **Figure 1** shows the division of India into six broad geographical regions based on the geographical locations and cultural settings [47]. The six broad geographical regions are:

North: Delhi, Haryana, Himanchal Pradesh, Jammu and Kashmir, Punjab, Rajasthan and Uttarakhand

Central: Chhattisgarh, Madhya Pradesh and Uttar Pradesh

East: Bihar, Jharkhand, Orissa and West Bengal

West: Goa, Gujarat and Maharashtra

South: Andhra Pradesh, Karnataka, Kerala and Tamil Nadu

Northeast: Arunachal Pradesh, Assam, Manipur, Meghalaya, Mizoram, Nagaland, Sikkim and Tripura

The bivariate analyses along with urban-rural (urban-rural) and gender (male-female) inequality ratios were calculated to understand differentials in child immunization across six broad geographical regions in India during 1992–2006. Chi-squared tests were applied in all six geographical regions to test the bivariate association. The urban-rural and gender inequality ratios were calculated as a ratio of full immunization rates:

Urban-rural inequality ratio (URIR)
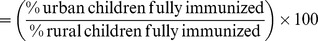



Gender inequality ratio (GIR)
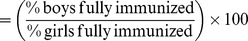



A value of 100 in an inequality ratio would imply that there was no urban-rural and gender differential in full immunization coverage, while a value above 100 would indicate inequality in the coverage of full immunization. Inequality in health is a multidimensional concept [48]. In a wider perspective, *“inequality in health”* is defined as inequalities in health that are unnecessary, avoidable, unfair and unjust [49]. The difference between place of residence (urban-rural) and gender (male-female) is presented in terms of relative inequality [50]. The term ‘inequality’ and ‘difference’ have been used interchangeably in the paper.

In order to examine the magnitude of change in full immunization coverage by urban-rural and gender across six geographical regions in India during 1992–2006, binary logistic regression models were applied after pooling the three rounds of NFHS datasets. A set of potential socioeconomic and demographic confounders were included to assess the net change in full immunization coverage by place of residence and gender. The three-way interaction between time with urban-rural and region and; time with gender and region were performed. Adjusted URIR and GIR were calculated to understand the net performance of the regions in full immunization during 1992–2006. As the sampling design of the three rounds of NFHS is comparable [51], many earlier studies have pooled the different rounds of NFHS datasets to observe changes over time [17], [51]. The results are presented as a set of predicted probabilities of being fully immunized, estimated using logistic regression post-estimation command available in Stata 10.0 [52]. The data analysis accounted for the multistage survey design using the “SVY” command in Stata.

## Results

### Trends in Child Immunization Coverage During 1992–2006

**Figure 2:**
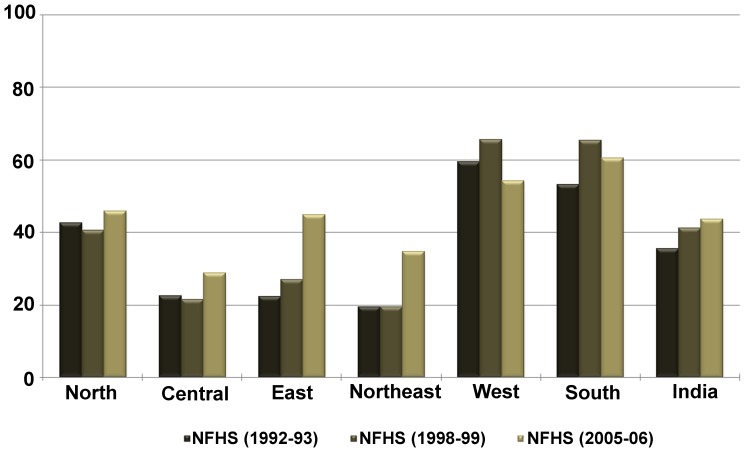
Trends in full immunization coverage (in %) among children aged 12–23 months by six geographical regions, India, 1992–2006.

**Figure 3:**
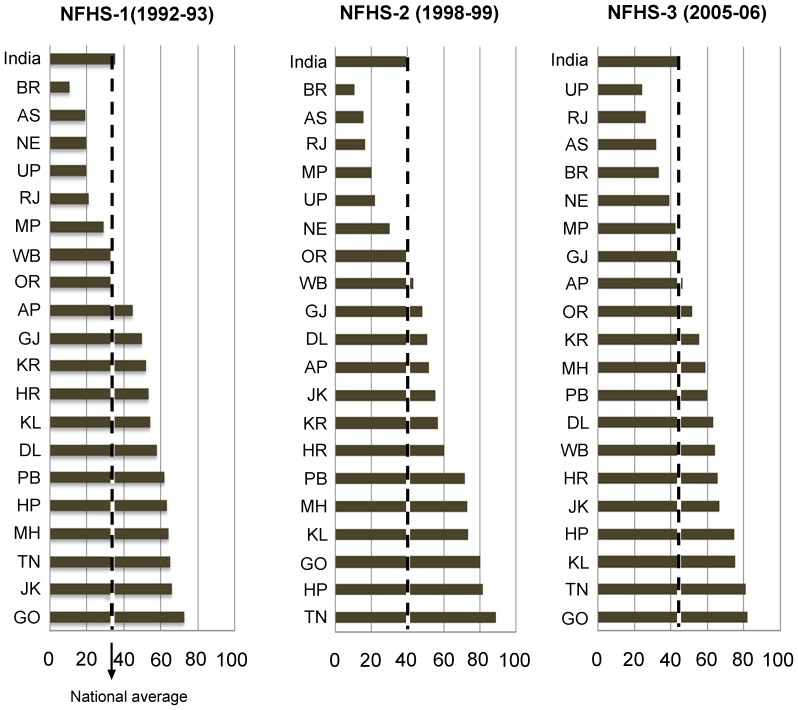
State wise full immunization coverage (in %) among children aged 12–23 months, India, 1992–2006. Notes: Abbreviations shown for the states are as follows: AP, Andhra Pradesh; BR, Bihar; GJ, Gujarat; GO, Goa; HP, Himachal Pradesh; HR, Haryana; JK, Jammu & Kashmir; KL, Kerala; KR, Karnataka; MH, Maharashtra; MP, Madhya Pradesh; OR, Orissa; PJ, Punjab; RJ, Rajasthan; TN, Tamil Nadu; UP, Uttar Pradesh; WB, West Bengal.

**Figure 2** shows at the national level, full immunization coverage had increased from 35% in 1992 to 44% in 2006. However, marked regional differences were observed during 1992–2006 in full immunization coverage, with the central region showing the lowest improvement. The state wise trends and patterns in full immunization coverage show in 1992–93, full immunization coverage in six states (West Bengal, Madhya Pradesh, Rajasthan, Uttar Pradesh, Assam, and Bihar) was lower than the national average of 35% (**Figure 3**). Although, the full immunization coverage increased to 44% in 2005–06, about 50% of the eligible children in nine states did not receive full immunization. The extent of inter-state difference in full immunization could be assessed as over 80% eligible children in Goa and Tamil Nadu utilized full immunization in 2005–06, while less than 25% of the children in Uttar Pradesh were fully vaccinated.

### Trends in Child Immunization by urban-rural and Gender across Six Geographical Regions

Noticeable variations in full immunization coverage were also reflected by urban-rural residence and gender across six broad geographical regions. The urban-rural inequality ratio (URIR) declined at the national level from 164 in 1992 to 149 in 2006 (**Table 2**). The regional trend shows that except in the west region, the URIR has declined during the last 15 years. On the other hand, the gender inequality ratio (GIR) increased from 108 in 1992 to 109 in 2006 at the national level (**Table 3**). The increase in GIR during the last fifteen years was also evident in the east, northeast, west and south regions. The highest increase in GIR was observed in the west (95 in 1992 to 112 in 2006), followed by the northeast (88 in 1992 to 98 in 2006) and the south region (104 in 1992 to 109 in 2006).

**Table 2:** Percentage of children (aged 12–23 months) who had received full immunization by place of residence in six geographical regions of India, 1992–2006, NFHS.

Region	1991–92		1998–99		2005–06		Urban-rural inequality ratio (URIR)^a^		
	Urban	Rural	Urban	Rural	Urban	Rural	1991–92	1998–99	2005–06
India	50.8	30.9	56.6	36.5	57.6	38.6	164.4	155.1	149.2
North	58.4	36.9	51.0	36.1	59.3	39.6	158.3	141.3	149.9
Central	36.3	19.6	36.3	18.1	45.4	25.2	185.6	201.0	180.0
East	34.7	19.7	43.7	24.5	56.4	42.5	176.2	178.4	132.8
Northeast	39.7	16.7	42.9	18.3	40.4	33.5	237.9	234.2	120.3
West	60.2	58.9	66.7	64.8	64.4	45.8	102.3	103.0	140.5
South	62.9	49.9	72.0	63.3	64.6	57.7	126.1	113.6	112.0

Note:

a Urban-rural inequality ratio calculated as (urban/rural*100).

Differences in full immunization by place of residence were significant at p<0.01 based on Chi-squared test in all three rounds of survey.

Differences in full immunization by region of residence were significant at p<0.01 in all the three rounds of survey based on Chi-squared test.

**Table 3:** Percentage of children (aged 12–23 months) who had received full immunization by sex of the child in six geographical regions of India, 1992–2006, NFHS.

Region	1991–92		1998–99		2005–06		Gender inequality ratio (GIR)^b^		
	Male	Female	Male	Female	Male	Female	1991–92	1998–99	2005–06
India	36.7	34.1	42.3	39.9	45.4	41.6	107.6	106	109.1
North	46.6	38.1	42.1	38.4	46.6	43.0	122.3	109.6	108.4
Central	25.4	19.3	25.1	17.9	31.2	27.4	131.4	140.2	113.7
East	22.5	21.8	27.2	26.3	45.7	43.5	103.5	103.4	105.0
Northeast	18.1	20.7	24.2	16.4	34.2	35.1	87.5	147.4	97.5
West	57.9	60.8	66.1	65.1	56.7	50.6	95.3	101.5	112.1
South	54.8	52.8	65.9	65.7	62.9	57.5	103.8	100.2	109.4

Note:

b Gender inequality ratio calculated as (male/female*100).

Differences in full immunization by sex of the child were significant at p<0.01 based on Chi-squared test in all three rounds of survey.

Differences in full immunization by region of residence were significant at p<0.01 based on Chi-squared test in all the three rounds of survey.

**Table 4** shows state wise trends in URIR and GIR in full immunization coverage across six geographical regions during 1992–2006. The overall pattern in URIR in full immunization shows that except two states –Tamil Nadu and Assam, full immunization coverage in all the remaining states was pro-urban in 2006. During 1992–2006, states like Assam (−142 points), Bihar (−84 points) and Rajasthan (−83 points) demonstrated a considerable decline in URIR. On the other hand, an increase in URIR was evident in Maharashtra (43), Madhya Pradesh (33), Kerala (22), Haryana (21), Gujarat (12) and Goa (4) during same period. As far as the trend in GIR is concerned, out of 20 major states, 12 states recorded pro-male full immunization coverage in 2006. The highest GIR in 2006 was recorded in Bihar (135), followed by Andhra Pradesh (133) and Punjab (122). During 1992–2006, the highest decline in GIR in full immunization was observed in Delhi (−30 points), followed by Rajasthan (−26 points) and Orissa (−23 points). However, the gender inequality ratio increased in Andhra Pradesh (26), Maharashtra (17) and Gujarat (11) during 1992–2006.

**Table 4:** Trends in gender and urban-rural inequality ratio of full immunization among children aged 12–23 months by state of residence, India, 1992–2006, NFHS.

Region/States	Urban-rural inequality ratio (URIR)			Absolute change in URIR	Gender inequality ratio (GIR)			Absolute change in GIR
	1992–93	1998–99	2005–06	1992–2006	1992–93	1998–99	2005–06	1992–2006
**India**	164.2	155.0	148.9	−15.2	107.7	105.8	109.1	1.4
**North**								
Jammu & Kashmir	136.0	136.7	111.8	−24.2	105.9	124.6	109.2	3.3
Himachal Pradesh	129.7	93.2	101.6	−28.1	113.2	108.8	101.5	−11.7
Punjab	131.9	127.5	112.1	−19.9	127.3	108.2	121.7	−5.6
Haryana	111.8	127.9	133.2	21.4	113.2	97.4	93.3	−19.9
Delhi	107.6	85.7	100.7	−6.9	129.7	126.3	100.0	−29.7
Rajasthan	282.5	201.4	199.3	−83.2	127.4	97.0	101.5	−25.8
**Central**								
Uttar Pradesh	189.0	174.7	160.8	−28.2	133.0	129.7	116.0	−17.0
Madhya Pradesh	168.6	262.2	201.8	33.2	128.4	161.3	114.5	−13.9
**East**								
West Bengal	140.4	140.5	112.7	−27.7	87.0	100.3	92.3	5.3
Orissa	125.8	142.4	102.4	−23.4	111.1	95.6	88.2	−22.9
Bihar	236.8	217.0	152.4	−84.3	142.2	130.5	134.8	−7.4
**West**								
Gujarat	123.0	120.8	135.2	12.2	106.1	106.2	117.1	11.0
Maharashtra	93.9	97.1	137.0	43.0	91.2	102.2	107.8	16.6
Goa	100.0	90.0	104.2	4.2	112.0	100.0	104.2	−7.8
**South**								
Andhra Pradesh	146.6	126.9	117.7	−28.9	107.3	87.4	133.2	25.8
Karnataka	115.4	86.8	113.1	−2.3	93.9	115.8	98.2	4.3
Kerala	103.4	103.5	125.2	21.8	105.4	95.4	96.4	−9.0
Tamil Nadu	121.3	113.4	92.6	−28.7	110.5	101.8	99.2	−11.3
**Northeast**								
Assam	235.6	288.9	94.0	−141.7	91.5	229.3	86.8	−4.7
Northeast (6 states)	259.0	163.9	135.5	−123.4	77.3	109.2	115.1	37.7

Note: Change in inequality ratio calculated as the difference in first and last survey (2006-1992).

### Regional Progression in Full Immunization and its Relation with urban-rural and Gender Inequality

The adjusted predicted probabilities obtained from the binary logistic regression analyses after pooling the datasets from three rounds of NFHS are presented in **Table 5** **.** The interactions between place of residence (urban-rural) and gender (male-female) in six geographical regions over time were statistically significant. This suggests that the urban-rural and gender inequalities with relation to full immunization across geographical regions have changed over time. Since, the present study focuses on urban-rural and gender inequality in full immunization coverage, more attention has been paid to prominent findings related to urban-rural and gender inequality.

**Table 5:** Predicted probability^a^ (PP) with 95% confidence interval (CI) of receiving full immunization among children aged 12–23 months across six geographical regions, India, 1992–2006.

Interactioneffect	India		North		Central		East		Northeast		West		South	
	PP	95% CI	PP	95% CI	PP	95% CI	PP	95% CI	PP	95% CI	PP	95% CI	PP	95% CI
**1992–93**														
Urban	0.529	[0.509–0.547]	0.584	[0.546–0.619]	0.443	[0.393–0.493]	0.364	[0.312–0.419]	0.420	[0.358–0.483]	0.631	[0.582–0.677]	0.610	[0.564–0.653]
Rural	0.300	[0.289–0.310]	0.318	[0.291–0.347]	0.221	[0.200–0.242]	0.221	[0.197–0.246]	0.151	[0.125–0.179]	0.536	[0.494–0.576]	0.482	[0.450–0.514]
Male	0.374	[0.360–0.388]	0.429	[0.396–0.461]	0.299	[0.271–0.328]	0.268	[0.236–0.301]	0.207	[0.171–0.247]	0.551	[0.506–0.506]	0.525	[0.488–0.561]
Female	0.339	[0.325–0.352]	0.342	[0.311–0.374]	0.245	[0.218–0.274]	0.242	[0.212–0.275]	0.204	[0.169–0.244]	0.573	[0.529–0.616]	0.508	[0.471–0.544]
Average^b^	0.348	[0.339–0.358]	0.392	[0.368–0.417]	0.270	[0.252–0.294]	0.255	[0.232–0.280]	0.211	[0.184–0.241]	0.559	[0.524–0.592]	0.517	[0.490–0.544
**1998**–**99**														
Urban	0.584	[0.562–0.605]	0.513	[0.470–0.555]	0.402	[0.342–0.464]	0.539	[0.472–0.603]	0.528	[0.457–0.597]	0.691	[0.643–0.734]	0.765	[0.719–0.805]
Rural	0.334	[0.321–0.346]	0.315	[0.286–0.344]	0.194	[0.171–0.220]	0.254	[0.227–0.283]	0.218	[0.186–0.252]	0.579	[0.531–0.625]	0.637	[0.600–0.672]
Male	0.411	[0.395–0.426]	0.388	[0.355–0.422]	0.276	[0.242–0.313]	0.323	[0.286–0.362]	0.308	[0.265–0.354]	0.604	[0.553–0.652]	0.678	[0.637–0.716]
Female	0.378	[0.362–0.394]	0.335	[0.301–0.370]	0.202	[0.172–0.235]	0.314	[0.275–0.354]	0.260	[0.218–0.305]	0.615	[0.564–0.662]	0.668	[0.626–0.706]
Average^b^	0.380	[0.368–0.340]	0.361	[0.336–0.388]	0.238	[0.252–0.294]	0.313	[0.286–0.342]	0.286	[0.253–0.321]	0.606	[0.569–0.640]	0.674	[0.490–0.702]
**2005**–**06**														
Urban	0.584	[0.565–0.601]	0.606	[0.555–0.655]	0.605	[0.564–0.644]	0.593	[0.543–0.640]	0.486	[0.437–0.535]	0.636	[0.592–0.677]	0.644	[0.603–0.682]
Rural	0.395	[0.380–0.408]	0.387	[0.348–0.427]	0.358	[0.326–0.391]	0.498	[0.460–0.535]	0.312	[0.277–0.348]	0.437	[0.388–0.486]	0.540	[0.495–0.583]
Male	0.477	[0.461–0.492]	0.471	[0.427–0.515]	0.460	[0.460–0.497]	0.529	[0.486–0.569]	0.398	[0.358–0.439]	0.530	[0.482–0.576]	0.604	[0.563–0.644]
Female	0.453	[0.437–0.469]	0.468	[0.422–0.514]	0.440	[0.402–0.478]	0.541	[0.498–0.582]	0.350	[0.310–0.391]	0.496	[0.445–0.546]	0.554	[0.508–0.598]
Average^b^	0.467	[0.455–0.479]	0.472	[0.438–0.505]	0.450	[0.421–0.480]	0.539	[0.508–0.571]	0.376	[0.344–0.408]	0.522	[0.486–0.557]	0.579	[0.547–0.610]
**Adjusted inequality ratios**														
URIR 1992–93	176.4		183.3		200.8		164.9		278.3		117.8		126.5	
URIR 1998–99	174.9		163.0		207.0		212.0		242.6		119.3		120.1	
URIR 2005–06	147.9		156.7		168.8		119.1		156.1		145.6		119.2	
GIR 1992–93	110.3		125.4		121.9		110.4		101.4		96.2		103.4	
GIR 1998–99	108.7		115.8		136.6		103.0		118.6		98.2		101.6	
GIR 2005–06	105.3		100.6		104.6		97.7		113.7		106.7		109.2	
**Relative percentage change**														
1992–1998	9.2		−7.9		−11.9		22.7		35.5		8.4		30.4	
1998–2006	22.9		30.7		89.1		72.2		31.5		−13.9		−14.1	
1992–2006	34.2		20.4		66.7		111.4		78.2		−6.6		12.0	

Note:

All predicted probabilities are significantly different at p<0.05 indicates the acceptance of alternative hypothesis in Wald test i.e., there was a significant difference in child immunization across six regions by place of residence (urban-rural) and gender (male-female) respectively during 1992–2006.

a Predicted probabilities adjusted for child wanted status, birth order & interval, mother’s age at birth, mother’s education, mother’s work status, mother’s mass media exposure, father’s education, religion, social groups and household wealth status.

b Adjusted by six regions along with other confounding variables mentioned previously.

URIR (urban-rural inequality ratio) calculated as: (urban/rural)*100 for each survey.

GIR (gender inequality ratio) calculated as: (male/female)*100.

Relative percentage change calculated as: (2006-1992)/1992*100.

Results show that the relative percentage change in immunization at the national level during 1992–2006 was 34 percentage points, while it was 9 percentage points during 1992–98 and 23 during 1998–2006. With regard to regional progression during 1992–2006, the highest percentage point change was estimated in the east region (111) and the lowest in the west (−7). The estimated trends in URIR and GIR based on predicted probability after adjusting the potential confounders show a declining pattern at the national level during 1992–2006. For instance, the URIR in India declined from 176 in 1992 to 157 in 2006. Similarly, in 1992 the GIR was 110, which declined to 105 in 2006. However, a mixed trend was evident in URIR and GIR across the six geographical regions in India. In the west region, the findings revealed an absolute increase of 28 points in URIR during 1992–2006, while in the remaining five regions the URIR declined over time, though at different pace. The highest decline in URIR of−122 points was evident in the northeast region, followed by the east (−46 points) and central (−32 points). In the case of GIR, the results suggest that out of six regions, in three regions namely, northeast, west and south, the GIR has increased between 1992 and 2006. The decline in GIR was as high as 12 points in the case of northeast, followed by west (11 points) and south (6 points). However, the highest decline in GIR was observed in the north region (25 points), followed by central (17 points) and east (13 points) regions.

## Discussion

Several programmatic efforts have been initiated including the Universal Immunization Program–1985, the Child Survival and Safe Motherhood Program–1992, the National Health Policy–2002, and the National Rural Health Mission–2005 to expedite the coverage of essential health services. However, this study shows considerable variation in childhood full immunization coverage across geographical regions in India over a period of one and a half decade (1992–2006). The results of this study have implications towards regulating child mortality in India, as about 31% of the deaths are due to infections, tetanus and other vaccine preventable diseases [5]. Despite a decline in urban-rural and gender differences in immunization coverage, children residing in rural areas and girls remain disadvantaged. The inequality trend in immunization coverage implies that the increase in average immunization may not essentially assure the universal and equitable coverage of immunization services, especially to the poor or underserved groups [53], in this case, rural children and girls.

Irrespective of the world regions, the urban advantage in the utilization of health care services over rural has been repeatedly emphasised [21], [54]. The urban infrastructure has often been attributed to the improved modern health care system [55]. In addition, there are many advantages for urban women including higher level of knowledge, access to services and health promotion programs that use urban-focused mass media [26]. In contrast, the utilization of health services in rural areas is limited by factors associated with availability, accessibility and quality of services as well as the characteristics of the users and the communities in which the users live [56]. Specifically, geographical access has a greater impact on the utilization of health care services, particularly in rural areas with limited service provision of health care services [57].

On the other hand, the persisting gender difference in full immunization across regions is strongly associated with the well evidenced practices of son preference in this part of the world. Several studies have examined the impact of gender preference on female foeticide [58], juvenile sex ratio [59], [60], number of ‘missing’ girls [61], [62], nutrition and healthcare [51], [63], [64], and increasing female mortality [30], [65]. In India or indeed in entire South Asia, sons are preferred over daughters for a number of economic, social and religious reasons, including financial support, old age security, property inheritance, dowry, family lineage, prestige and power, rituals and beliefs about religious duties and salvation [28], [59].

The present analysis demonstrates that regions with a higher rate of progression in full immunization showed a reduction in gender inequalities in immunization coverage during 1992–2006. However, regions such as northeast, west and south, which had a lower gender inequality in immunization coverage during 1992–93, observed an increase in gender difference over time, as the overall progression during the same period was relatively lower compared with other regions. This finding is in congruence with the recently conducted 2011 census that noted a decline in child sex ratio in many states [66]. Similarly, a recent trend analysis also documented an increase in coverage gap in health services (using a composite index of selected maternal, newborn and child health indicators) across rural and urban areas in states such as Gujarat, Haryana, Rajasthan and Kerala during 1992–2006 [67].

Regional differences in health outcomes have often been discussed in public health literature. Studies have shown the regional bias in the health outcome in India in terms of the ‘north-south dichotomy’ [65], [68], where majority of the north Indian states are characterised by low socioeconomic status, widespread poverty, low educational level, strong son preference, and low women’s autonomy [21], [61], [69]. However, the increasing gender bias over the period in full immunization coverage in better performing regions may question the “wrong region” hypothesis [63], which stated that neglect of the girl child is the outcome of being born in the wrong region (north, east and central). Similarly, the urban-rural differences in full immunization coverage have increased in the western region during 1992–2006.

The trend and pattern of inequality in child immunization across regions observed in this study could lead to the argument that the theory of “inverse equity hypothesis” has been persisting in India [70]. The hypothesis predicts that public health interventions will tend to increase inequity since they will initially reach those who are already better off. It is only when the wealthy have reached a level of improvement–beyond which interventions are unlikely to make more progress– that the poor begin to catch up and the inequity gap begins to reduce. Thus, only over time will the gap will be narrowed. The timing factor is therefore essential in the interpretation of the equity impact. Thus, the present study observes that rural and girl children received benefits in those regions, which observed an increase in average full immunization coverage during 1992–2006. However, urban-rural and gender difference has increased in regions where the average or overall progress in child immunization was lower during same period.

One of the strong arguments put forth for low immunization coverage is the failure of the ‘demand’ and ‘supply’ sides [13]. The demand failure includes the knowledge gap about the benefits of immunization, possibly due to lack of immunization campaigns and families not being adequately informed [71]. However, supply or system failure includes failure of the health worker to arrive on time or be regular, inadequate supplies and so on. Even though immunization is free in India, the travel costs along with waiting and opportunity costs can be high, particularly when it comes to the girl child and families residing in rural areas. This study confirms that the existing health inequity in India is due to the lack of attention given to social determinants of health highlighted by the WHO [72] and the Rio Political Declaration, 2011 [73], along with the malfunction of the health system to provide essential health services among those in need [53]. Moreover, programmatic initiatives primarily based on an assessment of average performance/achievement, rather than focus on within and between group differences, have rendered the vulnerable section, including rural and female children, more disadvantaged.

Recognizing the failure of previous programs, the Government of India launched the National Rural Health Mission (NRHM) in 2005 with the aim of providing effective health care to the rural population and providing equitable access to health facilities. However, the program has placed excessive emphasis on encouraging delivery care by providing cash incentives to women and health workers; this might overlook the other essential health services including immunization. There is evidence that community health workers have effectively reinforced immunization coverage in developing countries, particularly in rural and remote areas [74]. Strengthening the Integrated Child Development Schemes (ICDS), also highlighted in the Twelfth Five Year Plan (2012–2017) could play a vital role in ensuring improved coverage of immunization. Holding regular immunization camps at the community level and providing incentives have shown improvement in immunization rates [75].

### Potential Limitations of the Study

There are a few limitations that need to be acknowledged. The child immunization data has been collected from two sources: vaccination cards and mothers’ reporting. Using data from both mothers’ reports and immunization cards is more inclusive than from cards alone. Previous studies have evaluated the quality of information from mothers’ reports. Some of them underscore the high validity of mother’s recall, while other studies point to significant recall errors resulting in either overestimation or underestimation [76], [77]. In spite of potential sources of errors, the DHS (or NFHS) is considered as one of the best sources of population-based information on health and healthcare service utilization in low-and middle-income countries [78]. Thus, this study considers immunization coverage based on the DHS data to be of adequate quality and validity to which other measurement of the same variables can be compared. There are potential factors such as distance to immunization centres, quality of immunization services, behaviour and attitude of health personnel that could determine the full immunization coverage by residence and could act separately for male and female, which have not been included in this study due to data constraints.

## Conclusion

The High Level Expert Group (HLEG) on Universal Health Coverage in 2011 has emphasized equitable access to health services for all, alongside addressing wider determinants of health [79]. The HLEG has suggested the ‘National Health Package’ for essential health at the primary, secondary as well as tertiary levels for all citizens of India by 2022. This study emphasises that child immunization be included as a prioritized component in the proposed National Health Package, which could be an effective step towards ensuring universal immunization coverage. Integrating a strong immunization system, as part of a broader health system that closely coordinates with other primary health care delivery programs is essential for achieving the goal of universal immunization. In addition, there is need to integrate gender issues in India’s ongoing programmatic initiatives, particularly the immunization program, to which little attention has been paid.

The present study suggests that continued periodic evaluation of the performance of the health system is vital to assess the between and within groups difference. It is important to set aside the misconceptions that have prevented the utilization of health care services by certain regions/states in order to evaluate progress. The study also recommends targeted intervention among the marginalized sections of society and addressing obstacles in the way of utilizing health services. It is evident from the present study that those regions that have performed better conventionally, have neglected the growing inequality in immunization coverage due to less targeted interventions and lack of regular and systematic assessment. Moreover, further research is needed to identify comprehensive programmatic strategies to ensure sustainable immunization coverage, which was one of the major concerns during the 65th World Health Assembly held in 2012 [39].
